# Adenoid Cystic Carcinoma of the Submandibular Gland, Locoregional Recurrence, and a Solitary Liver Metastasis More Than 30 Years Since Primary Diagnosis

**DOI:** 10.1155/2014/581823

**Published:** 2014-10-27

**Authors:** A. Coupland, A. Sewpaul, A. Darne, S. White

**Affiliations:** ^1^Department of HPB and Transplant Surgery, Freeman Hospital, Newcastle upon Tyne NE7 7DN, UK; ^2^Department of Histopathology, Royal Victoria Infirmary, Newcastle upon Tyne NE1 4LP, UK

## Abstract

Adenoid cystic carcinoma (ACC) is a relatively rare tumour of the salivary glands, accounting for approximately 5%–10% of all salivary gland tumours. An important feature of ACCs is the long clinical course with a high rate of distant metastases. The preferential sites of metastases are the lung and bone, followed by the brain and liver. Most liver metastases are derived from nonparotid ACCs, and the presentation is often related to local recurrence or metastases to other organs. Solitary metastases to the liver are rare and optimal management is unknown. We present the case of a metastatic ACC to the liver with primary disease presentation at a young age. We discuss our management and other potential treatment modalities.

## 1. Introduction

Malignant salivary gland tumours are rare and adenoid cystic carcinomas (ACC) are one histological subtype in a heterogenous group [1, 2]. ACCs run a slow but progressive course and have a high likelihood of distant metastasis, most commonly to the lungs [3, 4]. Solitary metastases to the liver are rare and optimal management is unknown. We present the case of a metastatic ACC to the liver with primary disease presentation at a young age. We discuss our management and other potential treatment modalities.

## 2. Case Presentation

We report the case of a 52-year-old female who was referred to the surgical hepatobiliary team for assessment of a focal liver lesion found on a routine follow-up CT.

In 1975 (aged 15) the patient was diagnosed with an adenoid cystic carcinoma of the left submandibular gland. The primary lesion was excised followed by adjuvant chemotherapy and radiotherapy. Initial follow-up was for 10 years before discharge. In 2008 she represented with a lump at the left angle of mandible. A fine needle aspiration was suggestive of either benign pleomorphic salivary adenoma or recurrent ACC. A multidisciplinary team decision was made to treat the lesion conservatively. In 2010 the lesion had increased in size and a left total parotidectomy was attempted. Unfortunately, previous surgery and radiotherapy meant that only the superficial gland could be removed safely. Histological examination confirmed recurrence of salivary gland ACC.

A routine follow-up CT in February 2013 demonstrated a focal liver lesion that was suspicious of metastasis. No other metastases were found on further CT scans of the brain, head, neck, and chest. An MRI of the liver confirmed the presence of a solitary lesion (see Figure 2). A biopsy of the lesion confirmed the histological appearance of a metastatic adenoid cystic carcinoma that was of identical morphology to the parotid tumour excised in 2010. This had been performed prior to the patient being referred to our unit.

The patient was otherwise medically fit and did not have any other significant medical history. She did not take any regular medications.

**Figure 1:**
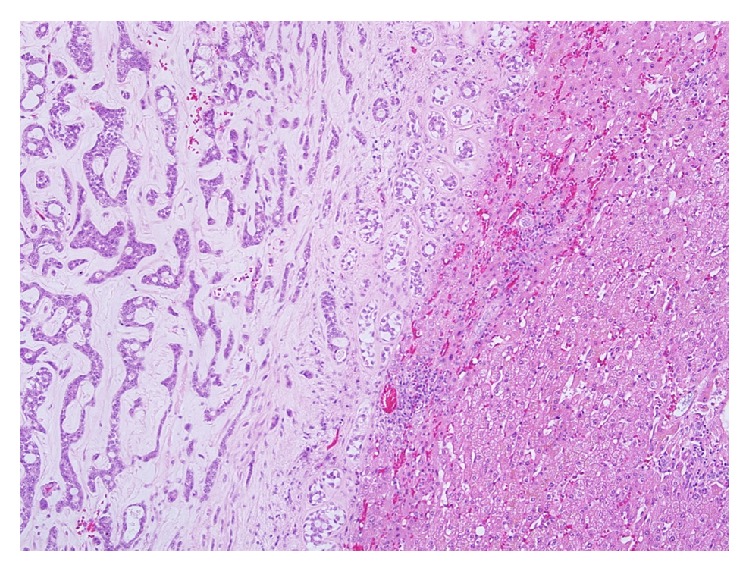
Tumour-liver parenchyma interface (×40). The tumour shows typical tubular and cribriform growth pattern with surrounding eosinophilic stroma.

**Figure 2:**
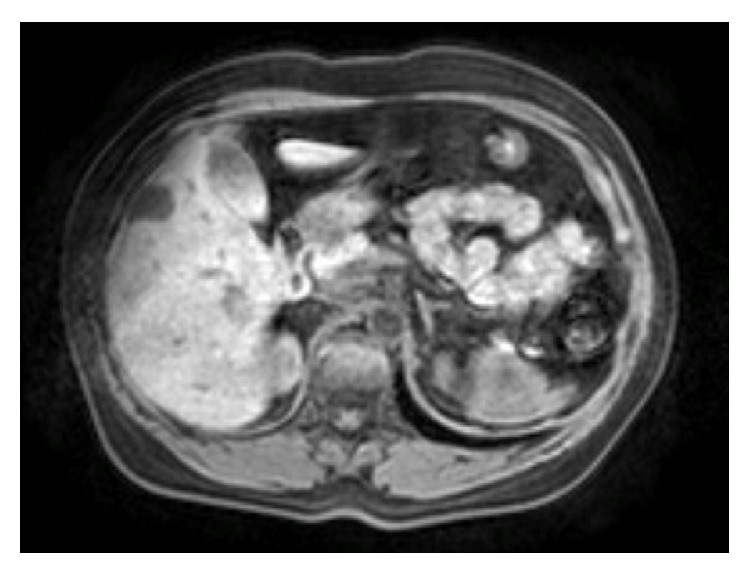
MRI demonstrating solitary segment V liver metastasis.

In June 2013 the patient underwent a nonanatomical segment V liver resection and cholecystectomy. The resected tumour measured 25 × 15 × 25 mm and demonstrated a 7 mm hepatic resection margin. The tumour was completely excised. No perineural, lymphatic, or vascular invasion was identified (Figure 1). The patient made an uncomplicated postoperative recovery and was discharged home.

**Figure 3:**
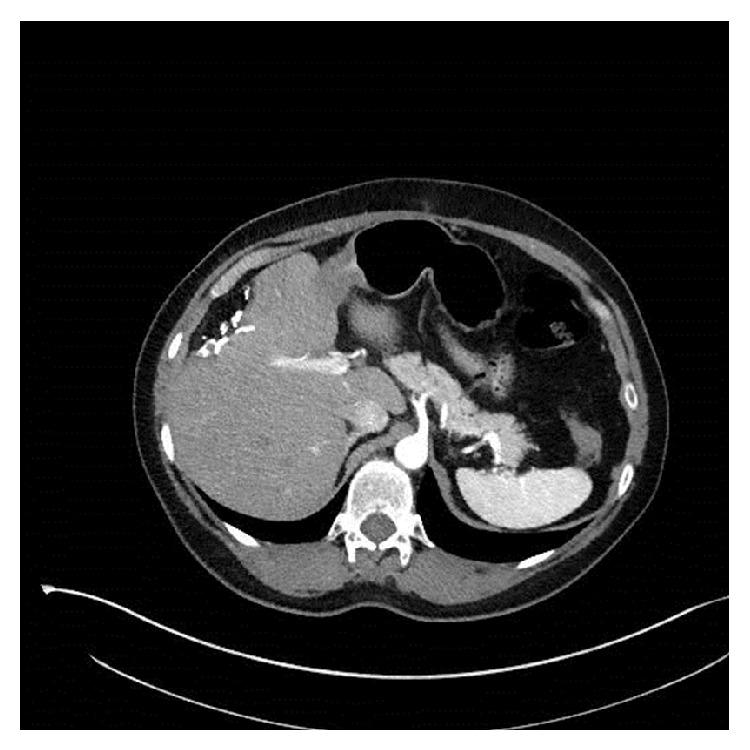
Axial view of follow-up CT scan showing resection margin and no evidence of recurrence.

**Figure 4:**
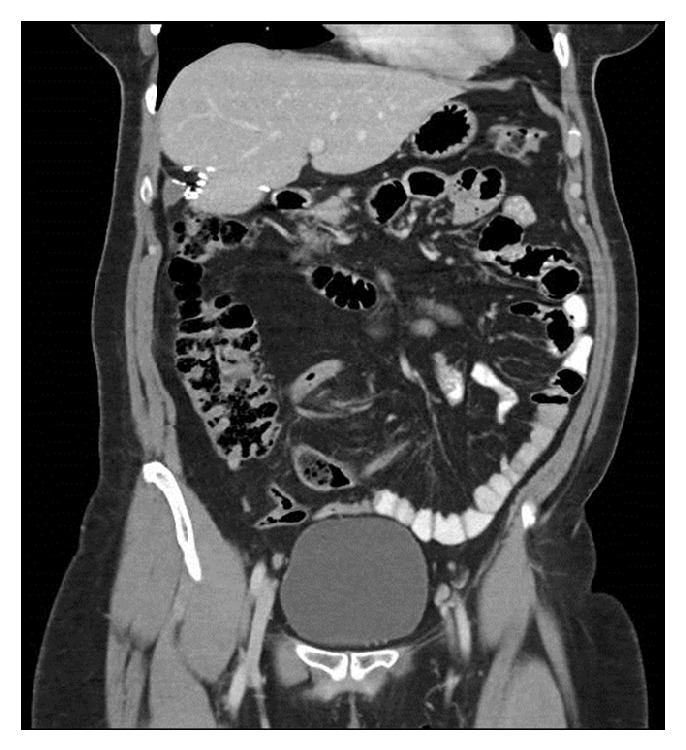
Coronal view of follow-up CT scan showing resection margin and no evidence of recurrence.

A 6-month follow-up CT of the neck/chest/abdomen and pelvis was performed as part of her follow-up. This did not demonstrate disease recurrence (see Figures 3 and 4).

## 3. Discussion

Carcinomas of the salivary glands are rare. Whilst it possible that our patient had a new primary arising from her salivary gland and not a recurrence of her initial primary this is less likely to be the case. A recent epidemiological study conducted in the United States, using the Surveillance Epidemiology and End Results (SEER) data, found that the overall incidence of major salivary gland carcinomas was 11.95 per 100 000 [1]. Amongst the broad histological heterogeneity of all salivary gland tumours, adenoid cystic carcinomas (ACC) account for approximately 10% [2]. The incidence of ACC is highest in the minor salivary glands [2]. There is an equal distribution between the submandibular and parotid glands (major salivary glands) unlike other histological subtypes of major salivary gland tumours, which have a higher incidence in the parotid [1].

It is documented that adenoid cystic carcinomas have a significant likelihood of delayed distant metastasis, even if locoregional control has been adequate [3]. Sung et al. [5] performed a retrospective clinicopathologic analysis of 94 cases of adenoid cystic carcinoma and found that, of the total cohort, 67% of patients had disease recurrence. 40% of these were due to the development of distant metastases alone (without locoregional recurrence) and the data concentrates on pulmonary and bone metastases. One patient developed liver metastases in conjunction with lung and bone metastases. Such a preponderance of lung metastases fits with other published data [3, 6], though metastases to other organs are recognised [6] and metastases to the liver greater in one series [3].

In data published by van der Wal [6] the average age of primary diagnosis of salivary gland ACC was 54.3 years and the average time between primary diagnosis and the detection of metastatic disease 36.8 months. This interval increases to 53.8 months when considering metastases to sites other than the lungs.

The average time between the detection of metastases (in sites other than the lungs) and death was 20.6 months according to van der Wal [6]. A short time interval between the diagnosis of metastases and death is also supported by Sung et al., who report that 11% of patients with distant metastases died within one year and 33% within 3 years [5].

The factors that statistically influence the development of distant metastases are histological growth patterns and primary tumour sites [5]. The major salivary glands are significantly more likely to metastasise than the minor salivary glands. In addition, those tumours of a solid histological growth pattern were more likely to result in distant metastatic spread than the cribriform or tubular histological subtypes [5].

Our case is primarily of interest because the primary diagnosis was made in a patient at such a young age (15 years old). Secondarily, the 35-year interval between primary diagnosis and locoregional recurrence and the 38-year gap before the diagnosis of distant metastases accurately represents the slow but relentless course of ACCs [2]. The case also contributes to a wider discussion regarding optimal treatment options for the metastatic ACCs to the liver.

Though rare, there is an increasing frequency of reported cases of ACCs either first presenting with hepatic metastatic disease [7–9] or of distant metastases that have arisen at variable times since primary diagnosis [10–12]. There is no consensus regarding optimal treatment. A number of treatment modalities have been published and the role of surgery has been questioned, as it may not prolong survival [8, 12]. However, there is a lack of randomised controlled trial data to support any course of management.

Balducci et al. [12] report a case with some similarity to the one reported here. Their patient was also found to have a solitary liver metastasis, although it arose 18 months following primary treatment. The patient received 3 cycles of chemotherapy with little response before undergoing an extended right hepatectomy and further adjuvant chemotherapy. The patient was disease-free for 18 months before final recurrence.

The role of chemotherapy has been questioned for metastatic ACC disease [13] and it is uncertain whether any lack of disease progression following systemic chemotherapy represents a true treatment effect or simply the indolent clinical course of metastatic ACC disease [14]. The role of drug-eluting bead chemoembolization, radiofrequency ablation, and surgical resection for distant metastases is uncertain, though Karatzas et al. [8] report a case that utilized each of the aforementioned treatment modalities for synchronous liver metastases. The patient remained disease-free for the 1 year of published follow-up.

There is little published on the outcome of liver resections for metastatic disease from ACCs, which likely represents disease rarity. In a multicentre analysis of noncolorectal, nonneuroendocrine hepatectomies for metastatic disease that included 420 patients, none of the metastases had an adenoid cystic carcinoma as a primary source [15]. It may be possible to extrapolate from this data that hepatectomies are technically feasible and safe for metastatic salivary gland disease based on the results from other malignant primary sources, but it is impossible to say whether resection has an impact on survival.

## 4. Conclusions

Our case is of interest because it highlights the paucity of data on how to manage the indolent and progressive course of metastatic salivary gland adenoid cystic carcinomas to the liver. We have contributed to the position that liver resections are feasible for hepatic metastases, but their significance in terms of disease-free and overall survival remains unknown. We support regular follow-up imaging and will need to consider the individual merits of multimodality treatments, such as chemoembolization and radiofrequency ablation for any recurrent hepatic disease.
